# Self-medication and non-adherence to antibiotic prescription and associated factors among Myanmar migrants in Thailand: a cross-sectional study

**DOI:** 10.1186/s12889-026-26443-z

**Published:** 2026-02-03

**Authors:** Hein Htet Zaw, Seo Ah Hong

**Affiliations:** 1 ASEAN Institute for Health Development, Mahidol University, Phutthamonthon, Nakhon Pathom Thailand; 2 Department of Public Health Administration, Faculty of Public Health, Mahidol University, 420/1 Ratchawithi Rd., Thung Phaya Thai, Ratchathewi District, Bangkok, 10400 Thailand

**Keywords:** Antibiotics, Self-medication, Non-adherence, Myanmar migrants, Thailand

## Abstract

**Background:**

Inappropriate antibiotic use has become a significant driver of the global burden of antimicrobial resistance (AMR). Our study aims to identify the proportion and associated factors of inappropriate antibiotic use, including self-medication of antibiotics, and non-adherence to antibiotic prescriptions among Myanmar migrants in Thailand.

**Methods:**

A cross-sectional study was conducted among 348 Myanmar migrants from three Myanmar migrant communities in Samut Sakhon, Thailand. The sample was recruited using convenience sampling, and the survey was conducted using face-to-face interviews. The variables, including predisposing, reinforcing, and enabling factors based on the PRECEDE-PROCEED model, were constructed. Descriptive statistics, Chi-square or Fisher’s Exact tests, and multiple logistic regression were performed to identify associated factors.

**Results:**

The proportion of self-medication among all participants was 28.4% (*n* = 348), and non-adherence among migrants who took prescribed antibiotics was 67.5% (*n* = 249). Factors positively associated with antibiotic self-medication are; never visiting a hospital in Thailand (AOR = 3.54, 95% CI = 2.00–6.27), inability to recognize common antibiotic drugs in the photos (AOR = 2.80, 1.56–5.02), and lack of AMR information in the past year (AOR = 1.98, 1.04–3.76). Factors positively associated with non-adherence to antibiotic prescription includes alcohol drinking (AOR = 37.58, 4.55–310.63), moderate to severe legal status-related acculturative stress (AOR = 6.49, 2.10–20.05 for severe stress and AOR = 2.60, 1.20–5.68 for moderate stress), receiving information regarding antibiotic use (AOR = 2.79, 1.40–5.58), working 7 days per week (AOR = 2.36, 1.12–5.01), lack of health insurance (AOR = 2.20, 1.17–4.14) and proximity to a hospital (AOR = 2.22, 1.15–4.28).

**Conclusion:**

High levels of inappropriate antibiotic use among Myanmar migrants highlight the need to integrate migrants into Thailand’s national action plan on AMR. The high prevalence of antibiotic misuse among Myanmar migrants highlights the need to include migrants in Thailand’s national plan to combat antimicrobial resistance. To improve patient understanding and adherence to treatment, healthcare professionals should provide medication guidelines and education on the risks of antimicrobial resistance. Policymakers should focus future policies and interventions on providing interpretation services and establishing institutional mechanisms to ensure migrants’ access to healthcare.

## Introduction

Antimicrobial resistance (AMR) has become a global public health threat. In 2021, antimicrobial-resistant bacterial infections may have caused 4.71 million deaths globally [1]. AMR places a significant financial burden, especially on low- and middle-income countries (LMICs) [2]. AMR’s health and economic burden in developing countries, including Thailand, far exceeds that of other priority diseases [3, 4]. AMR infections accounted for a direct treatment cost of over 6,000 million baht (approximately United States Dollar (USD) 172 million), and AMR-related premature deaths result in losses of at least USD 1.3 billion in Thailand [3, 4].

Since 2007, the Thai government has introduced the Antibiotic Smart Use Program (ASU) [5] to reduce unnecessary antibiotic prescriptions for minor illnesses, such as upper respiratory tract infections, acute diarrhea, and simple wounds. Additionally, the National Strategic Plan on AMR (2017–2021) set objectives to reduce antimicrobial consumption by 20%, decrease AMR morbidity by 50%, and increase public knowledge and awareness of appropriate antimicrobial use by 20% among Thai nationals [4, 6]. Although the causes of AMR are multifaceted, anthropogenic factors such as the overuse or misuse of antimicrobial agents, lack of treatment adherence, and increased disease transmission through global trade and travel also play a significant role in diminishing the effectiveness of antibiotics and accelerating the emergence of AMR [7–9].

Over 100 million people were displaced globally due to ongoing conflicts, climate change, and economic collapses during the last decade [10]. Thailand is also home to the second largest migrant population in Southeast Asia, primarily due to higher economic status, an aging society, and structural reliance on labor migration across many key economic sectors [11]. Especially migrants from neighboring countries like Myanmar, Cambodia, and the Lao People’s Democratic Republic are entering as the main labor force in Thailand [12]. As of December 2023, the total non-Thai population was over 5.3 million. At least 3 million Myanmar migrants were legally living in Thailand, and around 1.8 million were estimated as irregular Myanmar migrants [12, 13]. The geographically adjacent 2,400 km long Thailand-Myanmar border has already facilitated migration, a flow significantly accelerated by political turmoil following the 2021 military coup in Myanmar [13, 14]. Amid nationwide conflicts following the military coup, about 22,000 Myanmar nationals entered Thailand monthly for long-term stays in 2023, though 60% remained undocumented [13].

A systematic review study in Europe reported that migrant populations suffer from AMR infections more than the host population [15]. Migrants widely use antibiotic medicines inappropriately as first aid [16, 17]. The prevalence of unprescribed antibiotic use was reported to be 50% among migrants in Spain [16], which is higher than that of the host population (19%) [16]. A study in Jordan also reported 62% of self-medication among Palestinian refugees [17]. In Southeast Asia, including Thailand and Myanmar, one widely available form of self-medication is “Yaa Chud”, which is a small pack of medicines that contains various capsules and tablets, mainly antibiotics, steroids, and analgesics [18]. One of our previous studies revealed that about 45% of participants used Yaa Chud at least once in the past 3 months [19]. Meanwhile, there is a high prevalence of non-adherence to antibiotic treatment among adolescent migrants in the United Arab Emirates (38%) [20] and among Asian migrants in the Netherlands (45%) [21]. A study in Algeria found that 42.9% of Saharan refugees did not complete their course of medication [22]. Among the refugees in Bangladesh, medication adherence to antibiotic chemoprophylaxis for contacts of patients with diphtheria was only 55% at the follow-up visit [23]. A qualitative study in Iran revealed that study participants acknowledged the inappropriate use of antibiotics with low to medium levels of patients’ adherence to antibiotic therapy [24]. Many studies have shown that antibiotic treatment courses are incomplete due to difficulty with their use and unclear instructions [25].

Barriers to access to and appropriate use of antibiotics from the migrant’s side include poor access to health services and no health insurance. Because immigrants without health insurance have difficulty accessing the formal healthcare system, they must pay the hospital’s full cost of medication [26, 27]. They led migrants to self-medicate with antibiotics. In addition, they include language barriers, legal status, discrimination, financial burden, unsatisfactory experiences with health care services [28], easy availability of antibiotics in the community, satisfaction with prior self-medication [29], and lack of knowledge about appropriate antibiotic use. Knowledge and attitude toward antibiotics are also important factors associated with appropriate use. Most migrants have less knowledge than the host population, as found in studies done in European countries [30–32]. A survey of Thai adults showed that adequate knowledge and attitudes were associated with appropriate antibiotic use [33]. Migrants lacking knowledge about appropriate antibiotic use are more likely to perform self-medication as their first line of treatment rather than seeking care through formal healthcare services [26]. Attitude toward antibiotic use is significantly associated with antibiotic adherence [34]. Moreover, self-efficacy in the appropriate use of antibiotics refers to participants’ confidence in their ability to adhere to appropriate antibiotic use and overcome barriers. Self-efficacy in the appropriate use of antibiotics was high among the better educated [35]. Individuals with high self-efficacy are more likely to engage in self-medication [36]. Furthermore, alcohol consumption has adverse effects on adherence to medication [37]. Primary reasons may include forgetting to take doses due to alcohol intoxication or intentionally refraining from taking them to avoid drug-alcohol interactions [38, 39]. Yet, data on inappropriate antibiotic use among these vulnerable migrant populations are still scarce, particularly in Southeast Asia, including Thailand [40].

The increasing migrant population, along with the inappropriate use of antibiotics, such as self-medication and non-adherence, has become a significant contributor to the growing AMR problem nowadays. Particularly in LMICs, there are growing concerns about inappropriate antibiotic use due to poor antimicrobial surveillance. Despite higher rates of self-medication and non-adherence among immigrants than among the host population, national AMR strategies do not include immigrants. The exclusion of migrants from these national AMR strategies hinders efforts to address AMR in LMICs, including Thailand.

Therefore, addressing AMR requires a comprehensive understanding of inappropriate antibiotic use and its associated factors within migrant communities. Nonetheless, to our knowledge, there are very limited studies on the inappropriate use of antibiotics among growing Myanmar migrant populations in Southeast Asia, including Thailand. To our knowledge, our study is the first in Thailand to estimate the proportion of inappropriate antibiotic use due to self-medication and non-adherence to prescribed antibiotics, and to identify associated factors, among Myanmar migrants in Samut Sakhon, Thailand. Findings from our study on migrants’ antibiotic use behaviors and associated factors will provide valuable insights for the inclusion of migrant populations in the implementation of Thailand’s national action plans to combat AMR and support progress toward the health-related Sustainable Development Goals [12].

## Methods

### Study design and population

A cross-sectional study was conducted among Myanmar migrants in Samut Sakhon Province, Thailand, from August to September 2024. All Myanmar migrants aged 18 years or older who have been residing or working in Samut Sakhon Province for more than three months were included in this study. For sample size calculation, we used Cochran’s formula [41] with a 50% prevalence assumption of inappropriate antibiotic use among Myanmar migrants due to the lack of prior studies in Thailand or neighboring countries. To account for potential errors and missing responses, we added 10% (31 participants), resulting in a sample size 348. We purposively selected Mueang Samut Sakhon district, Samut Sakhon Province, Thailand, and three Myanmar communities with the largest Myanmar migrant population in the district due to the large population of Myanmar migrants living and working there [42]. We then recruited a total of 348 participants from the three communities via probability proportional to size (PPS) sampling: Thai Union (128 participants), Talad Kung (120 participants), and Talay Thai (100 participants). Our research team contacted Myanmar migrants with the support of community leaders and local Non-Governmental Organizations (NGOs) that support migrants in these areas.

The study questionnaire was initially developed in English based on previous studies and was reviewed by the Thesis advisory committee at Mahidol University to ensure content validity and clarity. It was then translated into Burmese by a bilingual medical doctor, followed by a back-and-forth translation process with Burmese medical doctors to ensure accuracy, cultural appropriateness, and linguistic clarity. Before data collection, pretesting of the questionnaire was conducted among 30 randomly selected Myanmar migrants with living conditions similar to those of the study population in Nakhon Pathom Province, Thailand, to ensure the questionnaire’s reliability. The Cronbach’s alpha values for different questionnaire constructs were acceptable (Adherence: 0.77, Attitude: 0.75, Self-efficacy: 0.852, acculturation stress: 0.63–0.87). After pretesting, minor Burmese words were modified based on expert opinions and migrant feedback from the pretest to improve clarity.

The semi-structured questionnaire was administered via a Google Form to facilitate data collection and cleaning. Data was collected in quiet, discrete community locations to ensure participant comfort and confidentiality. Due to potential literacy challenges among Myanmar migrants, the responses of participants who voluntarily participated in our study were collected through face-to-face interviews. The data were securely stored in the researcher’s online account.

### Study instrument

Based on the PRECEDE-PROCEED model [45], a conceptual framework was developed consisting of three parts: predisposing, reinforcing, and enabling factors. The semi-structured questionnaire included both closed- and open-ended questions.

Based on Wirtz et al. and the World Health Organization (WHO) [43], inappropriate antibiotic use behaviors of patients are defined as (i) self-medication of antibiotics (unnecessary use) in the entire sample and (ii) non-adherence to the prescription among those who received antibiotics with a prescription (incorrect use). Moreover, pharmacists in Thailand are legally authorized to dispense certain doses of antibiotics. Our study defined self-medication as the use of antibiotics obtained from sources other than a doctor, nurse, or licensed pharmacist [44, 45]. Participants were asked how they obtained antibiotics during their most recent episode of use, choosing from the following five options: (i) prescribed/given by doctors/nurses/licensed pharmacists, (ii) leftover antibiotics, (iii) purchased from grocery stores, street vendors, or hawkers, (iv) purchased online, or (v) others. If a participant selected an option other than the first, the response was classified as self-medication.

Adherence was measured using the Medication Adherence Report Scale (MARS-5) to categorize participants based on their medication-taking behavior with prescribed antibiotics. MARS-5 measured short-term antibiotic use adherence with a five-point Likert scale (5 = never, 4 = rarely, 3 = sometimes, 2 = often, and 1 = always) [46, 47]. Scores from the 5 items were summed to yield a total adherence score (0–25), with higher scores indicating greater adherence. Adherence score (≥ 23) and non-adherence score (< 23) were based on the standard classification used in the original MARS-5 validation study [48]. The Cronbach’s alpha for the Likert scale in our study was 0.77, indicating good reliability.

Predisposing factors include questions for socio-demographic characteristics such as age, gender, marital status, type of work, income, education, duration of stay in Thailand, health insurance, chronic disease status, knowledge on antibiotics use and resistance, recognition of drugs in the sample photo as antibiotics, ever heard about the term antibiotics, getting information about antibiotic unnecessary use and resistance within last year, attitudes toward antibiotic use and resistance, and self-efficacy to perform appropriate antibiotic use. The respondents’ ages were divided into two age groups using the mean age (28.7 years) as the cutoff point. Knowledge of antibiotic use and resistance was assessed using an adapted eight-question multiple-choice instrument (“TRUE,” “FALSE,” and “Don’t Know”) derived from a previous national study in Thailand [33]. Knowledge levels were categorized based on Bloom’s cut-off criteria, a widely used classification method in educational research. These thresholds (good: 80–100%, moderate: 60–79%, poor: <60%) align with benchmarks in previous related studies [49, 50]. Attitudes toward antibiotic use were measured using five Likert-scale questions (1 = strongly disagree to 5 = strongly agree), adapted from questionnaires used in Thailand’s national and WHO multi-country surveys [50, 51]. Total scores ranged from 5 to 25, with scores above the mean (20.6) indicating a positive attitude. Cronbach’s alpha for the Likert scale in the attitude section was 0.75 in our study. Self-efficacy to perform appropriate antibiotic use was measured using the Chinese version of the Appropriate Antibiotic Use Self-Efficacy Scale (AAUSES) [35]. The 13 items in the questionnaire assessed different self-efficacy domains using a 5-point Likert scale (0 = not confident at all to 4 = very confident), with total scores ranging from 0 to 52. The domains included are minimizing antibiotic use, trusting physician recommendations, avoiding antibiotics for viral infections, avoiding antibiotics based on prior medication experience, and avoiding antibiotics taken by others. Scores were categorized into three equal groups: low (< 15.2), medium (15.2–36.4), and high self-efficacy (> 36.4). The Cronbach’s alpha for self-efficacy questions was 0.85 in our study.

Reinforcing factors that can affect or provide motivation for migrants’ antibiotic use behaviors include recommendations from family and friends to use antibiotics, past satisfactory experiences with antibiotics, alcohol consumption behaviors, and acculturation stress. The 13-item acculturation stress scale, a modification of the Hispanic Stress Inventory [52], was adapted from a previous study of Mexican migrants [53]. The scale assessed stress due to perceived discrimination (items #1–4), language conflict (items #5–7), and legal residence status (items #8–13) on a 5-point Likert scale (1 = strongly disagree to 5 = strongly agree). Acculturation stress scores (range: 13–65) were categorized based on standard deviations (SD) from the mean (39.3 ± 12.5): low (< 26.8), moderate (26.8–51.8), and high (> 51.8). The same method was applied to categorize the three domain areas of the acculturation stress such as discrimination-related stress (low, < 8.2, moderate, 8.2–17.4, and high, > 17.4) (Cronbach’s alpha = 0.87), language-related stress (low, < 6, moderate, 6.0–12.8.0.8, and high, > 12.8) (Cronbach’s alpha = 0.63), and legal status-related acculturation stress (low, < 10.4, moderate, 10.4–24.0, and high, > 24.0) (Cronbach’s alpha = 0.80).

Enabling factors are the resources, skills, and environmental conditions that influence irregular antibiotic use among migrants. Our study included open-ended questions on the distance to health centers, travel time, and waiting time at health centers, and multiple-choice questions on satisfaction with healthcare services, perceptions of the cost of one healthcare visit, getting healthcare personnel’s explanation about diseases, and perceptions of the availability of antibiotics without prescriptions in the community.

### Statistical analysis

Responses from the online Google Form were downloaded in Excel format, and after data cleaning, data analysis was conducted using the Statistical Package for the Social Sciences (SPSS) version 21. Descriptive statistics were used to indicate the distribution of all variables in the study. Next, bivariate analyses were performed using the Chi-Square Test and Fisher’s exact test, as appropriate, to identify associations between independent and outcome variables. The logistic regression analysis included the significant independent variables with a p-value < 0.1. Before including them in the logistic regression analysis, multicollinearity was assessed using variance inflation factors (VIFs). No multicollinearity was detected (all VIFs < 5.0). Logistic regression analysis uses a backward stepwise method to build a logistic regression model by starting with all selected predictors and repeatedly removing the least significant variables until only statistically significant predictors remain. Hosmer and Lemeshow tests were used to assess the goodness of fit. Only the final significant variables with p-values less than 0.05 were extracted and displayed in the logistic regression results table. The adjusted odds ratio (AOR) was used to quantify the strength of association between the independent variables and the dependent variables. The confidence interval (CI) was used to quantify the precision of the OR estimate, and the p-value was used to assess the statistical significance of each association.

## Results

Table 1 presents general characteristics of the study respondents. More than half of the respondents were young people (aged 18–28.7 years) (59.5%), female respondents (60.3%), living alone (61.2%), and mainly employed in manual labor jobs such as seafood production and other manufacturing factories (79.0%), living in Thailand for less than 3 years (53.4%), earned less than 10,000 THB per month (68.1%), lacked health insurance (56.3%) and high education level (high school or higher education) (58%). Many Myanmar migrants already knew that antibiotics are germ-killing drugs (89.9%), correctly identified the drugs in the sample photo as antibiotics (60.3%), and had poor knowledge of antibiotic use and resistance (73.0%).

**Table 1:** Bivariate associations between predisposing factors and self-medication and non-adherence to antibiotic prescription

	Total		Self-medication of antibiotics without prescription (*n* = 348)				*p*-value	Non-adherence to antibiotic prescription (*n* = 249)				*p*-value
			No		Yes			No		Yes		
	*N*	%	*n*	%	*n*	%		*n*	%	*n*	%	
Total population			249	71.6	99	28.4		81	32.5	168	67.5	
Age group (Years) (Mean ± SD)	28.7 ± 9.7						0.216					0.891
18–28.7.7	207	59.5	143	69.1	64	30.9		30	21.0	113	79.0	
> 28.7	141	40.5	106	75.2	35	24.8		23	21.7	83	78.3	
Gender							0.249					0.217
Male	138	39.7	94	68.1	44	31.9		35	37.2	59	62.8	
Female	210	60.3	155	73.8	55	26.2		46	29.7	109	70.3	
Marital status							0.188					0.745
Living alone	213	61.2	147	69.0	66	31.0		49	33.3	98	66.7	
Living with a spouse	135	38.8	102	75.6	33	24.4		32	31.4	70	68.6	
Having children							0.094					0.258
No child	237	68.1	163	68.8	74	31.2		57	35.0	106	65.0	
One or more children	111	31.9	86	77.5	25	22.5		24	27.9	62	72.1	
Type of work							0.606					0.424
Manual labor workers	275	79.0	195	70.9	80	29.1		61	31.3	134	68.7	
Skilled workers and others	73	21.0	54	74.0	19	26.0		20	37.0	34	63.0	
Working hours per day (Mean ± SD)	8.1 ± 3.4						0.671					0.167
≤ 8 h	217	62.4	157	72.4	60	27.6		56	35.7	101	64.3	
> 8 h	131	37.6	92	70.2	39	29.8		25	27.2	67	72.8	
Working days per week (Mean ± SD)	5.5 ± 2.1						0.031					0.002
≤ 6 days	245	70.4	167	68.2	78	31.8		65	38.9	102	61.1	
7 days	103	29.6	82	79.6	21	20.4		16	19.5	66	80.5	
Monthly income (Mean ± SD)	9578.4 ± 4125.6						0.717					0.635
≤ 10,000 THB	237	68.1	171	72.2	66	27.8		54	31.6	117	68.4	
> 10,000 THB	111	31.9	78	70.3	33	29.7		27	34.6	51	65.4	
Education level							0.776					0.385
Low education level	147	42.2	104	70.7	43	29.3		37	35.6	67	64.4	
High education level	201	57.8	145	72.1	56	27.9		44	30.3	101	69.7	
Duration in Thailand (Mean ± SD)	6.5 ± 6.7						0.147					0.853
≤ 3years	186	53.4	127	68.3	59	31.7		42	33.1	85	66.9	
> 3 years	162	46.6	122	75.3	40	24.7		39	32.0	83	68.0	
Health insurance status							0.027					0.073
No Insurance	196	56.3	131	66.8	65	33.2		36	27.5	95	72.5	
Have Insurance	152	43.7	118	77.6	34	22.4		45	38.1	73	61.9	
Having chronic diseases (DM/Pulmonary diseases)							0.668					0.033^†^
No/Don’t know	333	95.7	239	71.8	94	28.2		81	33.9	158	66.1	
Yes	15	4.3	10	66.7	5	33.3		0	0.0	10	100.0	
Getting treatment for the above chronic diseases regularly							0.281					0.177^†^
No/Don’t know	341	98.0	244	72.0	95	28.0		81	33.2	163	66.8	
Yes	7	2.0	5	55.6	4	44.4		0	0.0	5	100.0	
Knowledge of antibiotic use and resistance							0.179					0.643^†^
Poor	254	73.0	175	68.9	79	31.1		59	33.7	116	66.3	
Moderate	87	25.0	68	78.2	19	21.8		21	30.9	47	69.1	
Good	7	2.0	6	85.7	1	14.3		1	16.7	5	83.3	
Recognition of drugs in the sample photo as antibiotics							< 0.001					0.489
As other drugs	138	39.7	78	56.5	60	43.5		23	29.5	55	70.5	
As antibiotics	210	60.3	171	81.4	39	18.6		58	33.9	113	66.1	
Being aware of the term “antibiotics"							< 0.001					1.000^†^
No	35	10.1	13	37.1	22	62.9		4	30.8	9	69.2	
Yes	313	89.9	236	75.4	77	24.6		77	32.6	159	67.4	
Getting information about the unnecessary use of antibiotics and resistance over the last year							0.002					0.017
No/Don’t know	247	71.0	165	66.8	82	33.2		62	37.6	103	62.4	
Yes	101	29.0	84	83.2	17	16.8		19	22.6	65	77.4	
Attitude towards antibiotic use and resistance							< 0.001					0.621
Poor attitude	131	37.6	79	60.3	52	39.7		24	30.4	55	69.6	
Good attitude	217	62.4	170	78.3	47	21.7		57	33.5	113	66.5	
Self-efficacy to perform the appropriate use of antibiotics							0.004					0.51
Low self-efficacy	58	16.7	32	55.2	26	44.8		22	68.8	10	31.3	
Medium self-efficacy	242	69.5	185	76.4	57	23.6		122	65.9	63	34.1	
High self-efficacy	48	13.8	32	66.7	16	33.3		18	56.3	14	43.8	

^†^ p-values from Fisher’s Exact tests

Regarding antibiotic use practices, 28.4% (99/348) of respondents reported self-medication. Regarding the self-medication, of predisposing factors (Table 1), chi-square test results revealed that working 7 days per week, having health insurance, recognition of drugs in the sample photo as antibiotics, being aware of the term “antibiotics,” getting information about antibiotic unnecessary use, and resistance within the last year, attitude towards antibiotic use and resistance, and self-efficacy were associated (*p* < 0.05). While of reinforcing factors (Table 2), there was no association between reinforcing factors and self-medication, among the enabling factors (Table 3), ever visiting hospitals/health centers in Thailand, distance to the nearest hospital, satisfaction with healthcare services, and receiving explanations from healthcare personnel about diseases were associated (*p* < 0.05).

**Table 2:** Distribution of respondents by symptoms for the last antibiotic use

Symptoms/Illnesses for last antibiotic use (multiple responses)	*n*	%
1. Sore throat	84	24.1
2. Cold and flu	150	43.1
3. Body aches	93	26.7
4. Toothache	11	3.2
5. Loose stool	14	4.0
6. Skin infection/wound infection	31	8.9
7. Passing white discharge/painful urination	10	2.9
8. Other reasons	28	8.0
9. Did not know or cannot remember the reason	49	14.1

**Table 3:** Bivariate associations between reinforcing factors and self-medication and non-adherence to antibiotic prescription

	Total		Self-medication of antibiotics without prescription (*n* = 348)				*p*-value	Non-adherence to antibiotic prescription (*n* = 249)				*p*-value
			No		Yes			No		Yes		
	*N*	%	*n*	%	*n*	%		*n*	%	*n*	%	
Recommendation from friends and family to use antibiotics when sick							0.610					0.504
No	109	31.3	76	69.7	33	30.3		27	35.5	49	64.5	
Yes	239	68.7	173	72.4	66	27.6		54	31.2	119	68.8	
Having satisfactory experience after self-medication with antibiotics							0.728					0.059
No	128	36.8	156	70.9	64	29.1		37	39.8	56	60.2	
Yes	220	63.2	93	72.7	35	27.3		44	28.2	112	71.8	
Failed to take antibiotics at the right times due to alcohol drinking							0.462					< 0.001^†^
Never	296	85.1	214	72.3	82	27.7		80	37.4	134	62.6	
Sometimes/Always	52	14.9	35	67.3	17	32.7		1	2.9	34	97.1	
Drinking alcohol while taking antibiotic medication							0.229					0.016^†^
Never	313	89.9	227	72.5	86	27.5		79	34.8	148	65.2	
Sometimes/Always	35	10.1	22	62.9	13	37.1		2	9.1	20	90.9	
Overall acculturation stress	39.3 ± 12.5						0.419					0.108
Low stress	63	18.1	43	68.3	20	31.7		23	53.5	20	46.5	
Moderate stress	219	62.9	162	74.0	57	26.0		106	65.4	56	34.6	
High stress	66	19.0	44	66.7	22	33.3		33	75.0	11	25.0	
Discrimination-related acculturation stress	12.8 ± 4.6						0.774					0.080
Low stress	79	22.7	54	68.4	25	31.6		29	53.7	25	46.3	
Moderate stress	200	57.5	145	72.5	55	27.5		102	70.3	43	29.7	
High stress	69	19.8	50	72.5	19	27.5		31	62.0	19	38.0	
Language-related acculturation stress	9.4 ± 3.4						0.989					0.739
Low stress	54	15.5	39	72.2	15	27.8		27	69.2	12	30.8	
Moderate stress	230	66.1	164	71.3	66	28.7		104	63.4	60	36.6	
High stress	64	18.4	46	71.9	18	28.1		31	67.4	15	32.6	
Legal status-related acculturation stress	17.2 ± 6.8						0.305					0.017
Low stress	72	20.7	53	73.6	19	26.4		28	52.8	25	47.2	
Moderate stress	223	64.1	154	69.1	69	30.9		100	64.9	54	35.1	
High stress	53	15.2	42	79.2	11	20.8		34	81.0	8	19.0	

^†^ p-values from Fisher’s Exact tests

**Fig. 1:**
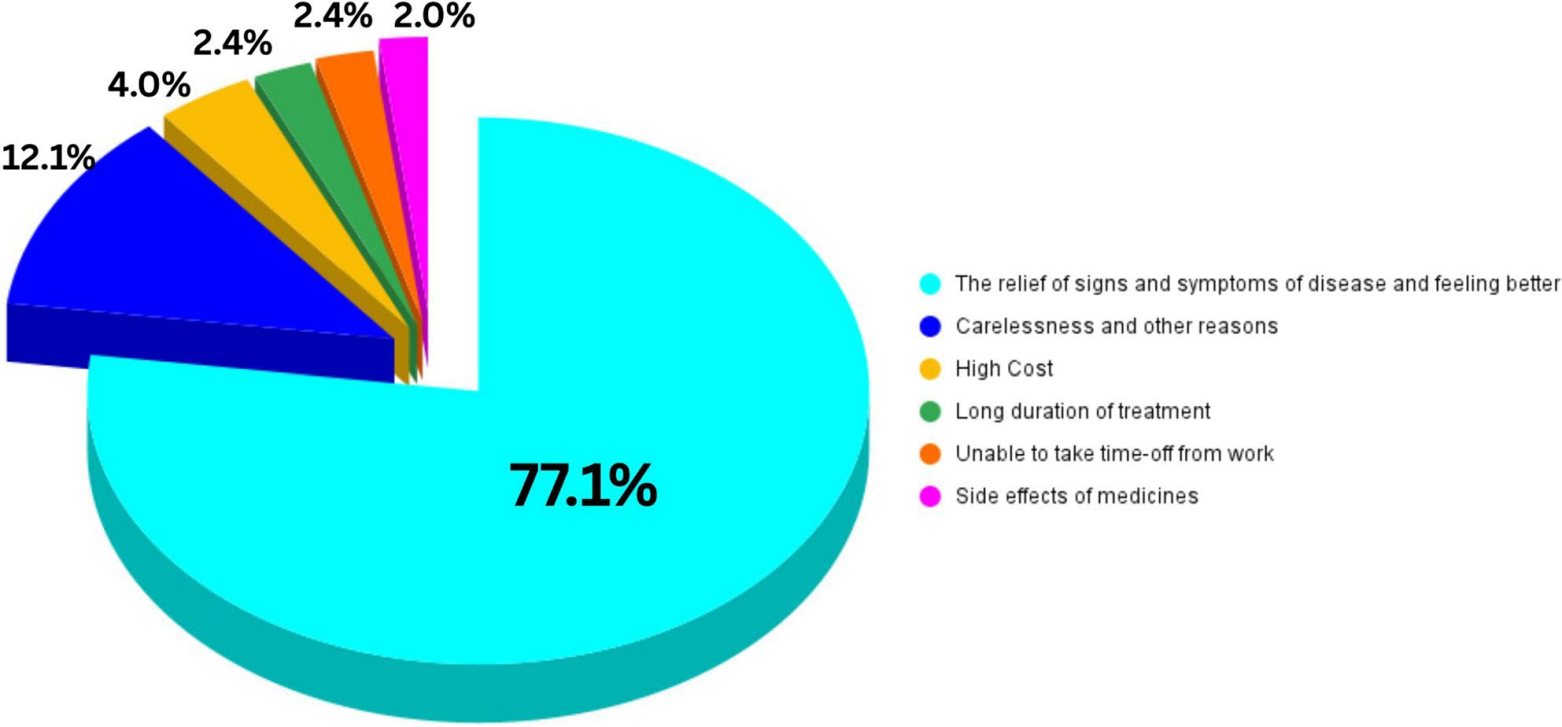
Reasons for stopping antibiotic use

Among the 249 respondents who received antibiotics from health personnel, 67.5% (168/249) demonstrated non-adherence to the prescription when assessed using the MARS-5 scale (Table 1). Colds and flu (43.1%), body aches (26.7%), and sore throat (24.1%) were the most common reasons for antibiotic use (Table 4). Notably, the reasons for stopping antibiotic use were mainly relief of signs and symptoms of diseases and feeling better (77.1%), followed by carelessness and other reasons (12.1%), high cost (4.0%), long duration of treatment (2.4%), being unable to take time off from work (2.4%), and side effect of medicines (2.0%)(Fig. 1).

**Table 4:** Bivariate associations between enabling factors and self-medication and non-adherence to antibiotic prescription

	Total		Self-medication of antibiotics without prescription (*n* = 348)				*p*-value	Non-adherence to antibiotic prescription (*n* = 249)				*p*-value
			No		Yes			No		Yes		
	*N*	%	*n*	%	*n*	%		*n*	%	*n*	%	
Ever been to hospitals/health centers in Thailand							< 0.001					0.424
No	96	27.6	54	56.3	42	43.8		20	37.0	34	63.0	
Yes	252	72.4	195	77.4	57	22.6		61	31.3	134	68.7	
Distance to the nearest hospital (Mean ± SD)	1.4 ± 2.7						0.043					0.020
≤ 1 mile	188	54.0	143	76.1	45	23.9		38	26.6	105	73.4	
> 1 mile/never been there	160	46.0	106	66.3	54	33.8		43	40.6	63	59.4	
Travel time to the nearest hospital	24.4 ± 15.1						0.176					0.023
≤ 20 min	157	45.1	118	75.2	39	24.8		30	25.4	88	74.6	
> 20 min/never been there	191	54.9	131	68.6	60	31.4		51	38.9	80	61.1	
Convenience with the duration of waiting time at hospitals							0.062					0.128
Not convenient/never been there	194	55.7	131	67.5	63	32.5		37	28.2	94	71.8	
Convenient	154	44.3	118	76.6	36	23.4		44	37.3	74	62.7	
Satisfaction with healthcare services							0.002					0.712
Not satisfied/never been there	243	69.8	70	60.9	45	39.1		24	34.3	46	65.7	
Satisfied	105	30.2	179	76.8	54	23.2		57	31.8	122	68.2	
Perception of the overall cost for one clinical visit							0.069					0.691
Cheap and affordable	185	53.2	140	75.7	45	24.3		47	33.6	93	66.4	
Expensive/never been there	163	46.8	109	66.9	54	33.1		34	31.2	75	68.8	
Getting healthcare personnel’s explanations about diseases							< 0.001					0.964
Explained	236	67.8	184	78.0	52	22.0		60	32.6	124	67.4	
Not explained or not understood clearly	112	32.2	65	58.0	47	42.0		21	32.3	44	67.7	
Availability of antibiotics without a prescription							0.982					0.607
Yes	193	55.5	111	71.6	44	28.4		43	31.2	95	68.8	
Not easy to buy/don’t know	155	44.5	138	71.5	55	28.5		38	34.2	73	65.8	
Ever taken Yaa Chud							0.176					< 0.001
No	142	40.8	96	67.6	46	32.4		44	45.8	52	54.2	
Yes	206	59.2	153	74.3	53	25.7		37	24.2	116	75.8	
Frequency of Yaa Chud taking in the past 3 months							0.715					0.013
No	195	56.0	138	70.8	57	29.2		54	39.1	84	60.9	
1 or more times	153	44.0	111	72.5	42	27.5		27	24.3	84	75.7	

Regarding non-adherence to antibiotic use, of predisposing factors (Table 1), working 7 days per week, having chronic diseases, and receiving information about unnecessary antibiotic use and resistance in the last year were associated (*p* < 0.05). Of reinforcing factors, failure to take antibiotics at the appropriate times due to alcohol drinking sometimes or always, drinking alcohol while taking antibiotic medication, and legal status-related acculturation stress were associated (*p* < 0.05)(Table 2) and of the enabling factors, distance and travel time to the nearest hospital, ever taking Yaa Chud, and frequency of Yaa Chud in the past 3 months were associated (*p* < 0.05)(Table 3).

The multivariable analysis results showed that three variables were significantly associated with self-medication. Self-medication was more likely among respondents who could not recognize sample antibiotics in the photo (AOR = 2.80, 95% CI = 1.56–5.02), did not get information about antibiotic unnecessary use and resistance within last year (AOR = 1.98, 95% CI = 1.04–3.76), and had never visited a hospital in Thailand (AOR = 3.54, 95% CI = 2.00–6.27) (Table 5).

**Table 5:** Results of multiple logistic regression of factors associated with self-medication of antibiotics without prescription (*n* = 348)

Variables	Self-medication of antibiotics (*n* = 348)			
	AOR	95% CI of AOR		*p*-value
		Lower	Upper	
Recognition of drugs in the photo as antibiotics				
No	2.8	1.56	5.02	0.001
Yes	1			
Getting information about the unnecessary use of antibiotics and resistance within the last year				
No/Don’t know	1.98	1.04	3.76	0.037
Yes	1			
Ever been to hospitals/health centers in Thailand				
No	3.54	2	6.27	< 0.001
Yes	1			

The variables with p-value < 0.1 in Chi-square results included having children, working days per week, health insurance status, knowledge level about antibiotics and antibiotics resistance, knowing the drugs in the photo as antibiotics, ever heard about the term antibiotics, getting information about antibiotic unnecessary use and resistance, attitude towards antibiotics use and resistance, self-efficacy to perform appropriate use of antibiotics, ever visited hospitals or health centers in Thailand, the distance to nearest healthcare center, being convenient with waiting time at healthcare centers, having satisfactory experience with healthcare services, perception on the cost of one hospital visit and healthcare personnel explanation about diseases at the hospital

The results were from the Backward elimination method

Hosmer and Lemeshow’s test results indicated a good fit (Χ2 = 2.391, P = 0.967)

For non-adherence to prescribed antibiotics among respondents who received a prescription (Table 6), significantly associated factors were working 7 days a week (AOR = 2.36, 95% CI = 1.12–5.01), lacking health insurance (AOR = 2.20, 95% CI = 1.17–4.14), and getting information about antibiotic unnecessary use and resistance within the last year (AOR = 2.79, 95% CI = 1.40–5.58). Additionally, shorter travel time to the nearest healthcare facility (≤ 20 min) (AOR = 2.22, 95% CI = 1.15–4.28), failure to take antibiotics at right times due to alcohol drinking sometimes or always (AOR = 37.58, 95% CI = 4.55–310.63), and experiencing moderate or severe legal status-related acculturation stress (AOR = 2.60, 95% CI = 1.20–5.68 and AOR = 6.49, 95% CI = 2.10–20.05, respectively) were also significant predictors of non-adherence.

**Table 6:** Results of multiple logistic regression of factors associated with non-adherence to antibiotic prescription among those who took antibiotics with a prescription (*n* = 249)

Variables	Non-adherence to antibiotic prescription (*n* = 249)					
	AOR	95% CI of AOR				*p*-value
		Upper		Lower		
Working days per week						
≤ 6days	1					
7 days	2.36	1.12		5.01		0.025
Health insurance status						
No Insurance	2.20	1.17		4.14		0.014
Have Insurance	1					
Getting information about the unnecessary use of antibiotics and resistance in the last year						
No/Don’t know	1					
Yes	2.79	1.40		5.58		0.004
Failed to take antibiotics at the right times due to alcohol drinking						
Never	1					
Sometimes/Always	37.58	4.55		310.63		0.001
Legal status-related stress						
Low stress	1					
Moderate stress	2.60	1.20		5.68		0.016
High stress	6.49	2.10		20.05		0.001
Time taken to travel to the nearest hospital						
≤ 20 min	2.22	1.15		4.28		0.018
> 20 min	1					

The variables with p-value < 0.1 in Chi-square results included working days per week, working hours per week, health insurance status, having chronic diseases (such as diabetes mellitus and/or chronic pulmonary disease history), getting information about antibiotic unnecessary use and resistance, having satisfactory experience with self-medication, failure to take antibiotics at the right times due to alcohol drinking, drinking alcohol together with the antibiotic medication, discrimination stress level, legal status related acculturation stress, the distance to nearest health center, time taken to travel to the closest healthcare center, having experience of taking Yaa Chud drugs, frequency of Yaa Chud taking in past 3 months. The results are from the Backward method

Hosmer and Lemeshow test results indicated a good fit (Χ2 = 4.007, P = 0.857)

## Discussion

Our study aimed to identify inappropriate antibiotic use, including self-medication and non-adherence, among Myanmar migrants in Thailand. Our study showed that about one-third of Myanmar migrants reported self-medication with antibiotics, while 67.5% who obtained antibiotics with a prescription did not adhere to the prescribed therapy. Moreover, most of the Myanmar migrants in our study exhibit poor knowledge of antibiotic use and antibiotic resistance. These findings underscore the importance of including migrants in policies and practices to effectively implement Thailand’s national action plan to combat AMR.

### Proportion of self-medication

Our results showed that nearly one-third of Myanmar migrants used antibiotics obtained from sources other than licensed health personnel in Thailand. The prevalence is higher than that in the Thai adult population (29.7%) [33]. Interestingly, the prevalence of self-medication among migrant populations in other countries is even higher, including Australia (41%) [54], Spain (50%) [55], and Jordan (62%) [17]. The variation in prevalence across countries may be attributable to differences in regulatory frameworks and cultural beliefs regarding antibiotic access. For example, antibiotics are prescription-only drugs in countries such as Australia, where pharmacists are not legally permitted to dispense antibiotics without a valid prescription from a general practitioner [56]. In Myanmar, a national study in 2020 revealed that more than half of the study population (58.5%) consumed antibiotics without a prescription, where this widespread practice was attributed mainly to the country’s relatively lax pharmaceutical regulations and cultural beliefs on antibiotics as a fast-acting drug for illness [57]. Meanwhile, the relatively low rate of antibiotic self-medication among Myanmar migrants is likely because, in our study, antibiotics dispensed by licensed pharmacists were not considered self-medication under Thailand’s pharmaceutical laws [45, 58]. A survey among community pharmacists in Thailand revealed that community pharmacists have good knowledge and attitude towards antibiotic prescription and AMR [44], which may be partly due to the implementation of the ASU project [5] and Thailand’s National Strategic Plan on Antimicrobial Resistance (2017–2022) [6], as well as new pharmacy laws and regulations such as the Good Pharmacy Practice in community pharmacy [59], the continuing pharmacy education [60] in Thailand.

### Factors associated with self-medication of antibiotics

Our multiple logistic regression results showed that, of predisposing factors, Myanmar migrants who had never received information about unnecessary antibiotic use and antibiotic resistance in the last year and who did not recognize the sample antibiotic drugs in the photo were more likely to self-medicate with antibiotics. Previous studies among Chinese migrants also stated that poor antibiotic-related knowledge and misperception of antibiotic use were significantly associated with self-medication of antibiotics [61]. The 2020 national survey in Myanmar also reported low levels of knowledge and awareness about antibiotic use and the high prevalence of antibiotic self-medication among the general population [57]. In our study, the most common reasons for antibiotic use were colds and flu (43.1%), body aches (26.7%), and sore throat (24.1%), indicating a misconception among Myanmar migrants that antibiotics are used for pain relief. These results underscore the critical need for targeted interventions to improve antibiotic knowledge and awareness among Myanmar migrants in Thailand, particularly those with limited access to formal healthcare. Furthermore, the odds of self-medication of antibiotics without prescription were higher among respondents who had never been to hospitals/health centers in Thailand. A previous multisectoral assessment report on Samut Sakhon Province, Thailand, also reported that 32% of migrants faced barriers to accessing formal medical services [42]. Similarly, Myanmar migrants with incomplete legal documents usually tend to avoid visiting health centers or hospitals and perform self-medication due to the fear of deportation or being reported to the police at health centers in Thailand [62] and also full medication charges at the hospitals if they don’t have health insurance, even at the community hospitals in Thailand. Previous studies have reported that difficulties accessing the formal healthcare system and obtaining health insurance in host countries have led migrants to prefer self-medication [26, 63–67]. This lack of knowledge, along with barriers to accessing formal healthcare services, creates a perfect storm for self-medication among this vulnerable population. Therefore, migrant-specific community-based antibiotic awareness programs or pharmacist-led interventions are recommended to improve antibiotic knowledge and awareness among Myanmar migrants in Thailand. The Thai government and NGOs should also promote accessibility to health insurance and the Thai healthcare system for migrants to prevent unnecessary antibiotic use in Thailand.

### Proportion of non-adherence

Inconsistent with previous studies reporting that immigrants and refugees have higher rates of non-adherence to medication compared to native groups [70], our study showed that 67.5% of 249 Myanmar migrants who took antibiotics with a prescription were not adherent to the prescription. A survey among Asian immigrants in the Netherlands also showed 45% of non-adherence to antibiotic therapy [21]. A systematic review conducted across countries in the WHO Southeast Asian Region also reported that the most common inappropriate antibiotic use practice was stopping the antibiotic use immediately after symptom resolution [68]. Our finding supports the conclusion that the primary reasons for stopping antibiotic use were relief of signs and symptoms of disease and feeling better (77.1%), consistent with previous studies [61, 69]. Therefore, the high prevalence of non-adherence among the migrants reflects the need for targeted intervention to improve medication adherence to antibiotics among them.

### Factors associated with non-adherence

Regarding predisposing factors, our study found that migrants working 7 days a week were less likely to adhere to antibiotic prescriptions. It is explained that being unable to take time off work was one of the reasons for stopping antibiotic use in our study. Research also stated that exploitations in working hours, sick leaves, and mandatory leaves were barriers to using healthcare services in hospitals and healthcare centers for undocumented Myanmar migrants in Samut Sakhon Province, Thailand [70]. A survey among Myanmar migrants in the Thailand-Myanmar border area stated that prioritizing work over regular medication use was a significant reason for medication non-adherence [71]. Moreover, prioritizing work over treatment adherence often outweighed the influence of facilitating factors such as health literacy, health education, and social norms [71]. Therefore, including mandatory regulations on working hours and holidays in migrant labor rights and policies is essential to enhance formal access to healthcare services and improve medication adherence among Myanmar migrant workers. Furthermore, non-adherence to antibiotic prescriptions among respondents without health insurance was higher than among those with health insurance in Thailand. A previous systematic review stated that financial burden and lack of health insurance were associated with treatment adherence in the immigrant and refugee populations [72]. Undocumented or irregular migrants may exhibit poor adherence to prescribed antibiotics, even when frequent clinic visits are not required. For migrants with insecure or unstable immigration status, fear of arrest and unemployment may lead immigrant workers to prioritize work performance over treatment compliance [71], which may result in non-adherence to medication.

Interestingly, our study found that those living within 20 min of a healthcare facility and getting information about unnecessary antibiotic use and resistance over 12 months were less likely to adhere to antibiotic prescriptions. For immigrants who prioritize work over treatment due to legal vulnerability and fear of unemployment, it may be interpreted that access to healthcare facilities or information alone does not increase the likelihood of compliance with antibiotic prescriptions. It may be more evident for those without health insurance. Migrant workers in low-wage sectors without health insurance may not be able to afford or give priority to continue taking medicines as prescribed once their symptoms are relieved. Moreover, this may be due to the cultural belief in the Myanmar people that antibiotics are fast-acting drugs [73], as well as the easy access to antibiotics in pharmacies in Myanmar and Thailand, which may have influenced the casual attitude toward antibiotics [74, 75]. Moreover, high legal-status-related acculturative stress was associated with non-adherence to antibiotic medication in our study. It may be supported by a previous study among Latino migrants, which found that better acculturation in the host country is associated with better medication adherence [76]. Therefore, the fear of deportation, stigma, and discrimination among Myanmar migrants due to high acculturation stress should be addressed by integrating the mental health support programs for migrants in AMR interventions. Therefore, awareness-raising activities on the importance of adherence to antibiotic prescriptions and on AMR in Thailand should be conducted in collaboration with relevant stakeholders, including migrant health volunteers, NGOs, and community leaders. In addition, it is necessary to support the documentation process to facilitate the enrollment of Myanmar migrants in Thailand’s health insurance and to establish fundamental labor rights and work schedules for Myanmar migrant workers, thereby improving migrants’ access to the formal healthcare system in Thailand, while also ensuring the provision of quality services at reasonable prices.

Respondents who sometimes or always failed to take antibiotic doses at appropriate times because of drinking alcohol were more likely not to follow antibiotic treatment guidelines than those who never drank alcohol in our study. This finding is consistent with that of Bryson CL et al. (2008) [77], who also found that an increase in alcohol misuse is associated with an increased risk for medication non-adherence among patients in a primary healthcare center. Similarly, heavy episodic drinkers (≥ 5 drinks in one day in the past 30 days) and non-episodic drinkers were slightly more likely to exhibit poor medication adherence than abstainers [78]. Another study showed that medication adherence was significantly lower on days when individuals drank more than their typical amount of alcohol [79]. The results of this study provide further support for the hypothesis that alcohol drinking can affect the appropriate use of antibiotics among migrants in Thailand. The large AOR and wide 95% CI were likely due to the very few events [80], as most respondents in our study were female (60.3%), who typically do not drink alcohol in the Myanmar migrant community. Furthermore, the questions on alcohol-related behaviors may be subject to recall bias and social desirability bias since some answers depended on their perception rather than the actual condition that happened. Therefore, caution is needed when interpreting these results. Further studies using a more representative sample of male immigrants is needed to investigate alcohol-related factors in the inappropriate use of antibiotics.

Our study’s limitation primarily stems from its cross-sectional design. Using a cross-sectional study design requires caution when interpreting results, as it precludes causal inferences. Therefore, longitudinal studies are recommended to track antibiotic use patterns over time. Moreover, the convenience sampling used in our study limits generalizability and may introduce selection bias relative to stratified random sampling. However, the informal nature of the Myanmar migrant population makes it challenging to reach highly mobile populations, i.e., migrants. Convenience sampling is thus practical for achieving the expected sample size within the time, resource, and accessibility constraints of the survey. Therefore, we deliberately selected the three Myanmar migrant communities with the largest populations and recruited migrants from these communities. Further research should employ random sampling at the community or household level to ensure a more representative sample and enhance the accuracy of findings. In addition, because our study definition of self-medication excludes antibiotics dispensed by pharmacists, which aligns with Thai pharmaceutical laws, this may underestimate the true prevalence, as many migrants with language barriers and low literacy may not know or recall whether the antibiotic dispenser was a valid, licensed pharmacist. Such nondifferential misclassification can often lead to information bias that underestimates the true effect of exposure on outcomes. Lastly, respondents were asked about their prior experience with antibiotic use during episodes of illness and their perceptions of healthcare center utilization in Thailand. This self-reported nature of data introduces recall and social desirability bias. Despite these outlined limitations, our study was the first to explore inappropriate antibiotic use behaviors and associated factors among the Myanmar migrants residing in Thailand.

## Conclusion

Our study found that nearly one in three Myanmar migrants engaged in antibiotic self-medication, and approximately 70% of those who used prescribed antibiotics did not adhere to the regimen. This finding suggests that addressing the problem of inappropriate antibiotic use among Myanmar migrants is essential for effectively achieving the goals of Thailand’s National Strategic Plan on Antimicrobial Resistance. In addition, for immigrants who prioritize work over treatment due to legal vulnerability and fear of unemployment, access to healthcare facilities or information alone does not increase the likelihood of compliance with antibiotic prescriptions. Healthcare providers should provide medication guidelines and educate patients about the risks of antibiotic resistance to improve patient understanding and adherence to treatment. Public health policymakers should design and implement culturally sensitive public health programs at the health center and community levels to educate Myanmar migrants about the risks of antibiotic self-medication and antibiotic resistance. They should also prioritize the provision of interpretation services and the establishment of institutional mechanisms to ensure migrants’ access to healthcare.

## Data Availability

All data generated or analyzed during this study are included in this article.

## Abbreviations

AMR: Antimicrobial resistance
AOR: Adjusted Odds Ratio
ASU: Antibiotic Smart Use program
CI: Confidence Interval
DM: Diabetes Mellitus
LMICs: Low- and middle-income countries
MARS: Medication Adherence Report Scale
NGO: Non-Governmental Organization
SD: Standard Deviation
USD: United States Dollar
WHO: World Health Organization

## Declarations

### Content for publication

It is not applicable because our manuscript does not contain any of the person’s data (including individual details, images, or videos).
